# Bystander effectors of chondrosarcoma cells irradiated at different LET impair proliferation of chondrocytes

**DOI:** 10.1007/s12079-019-00515-9

**Published:** 2019-03-22

**Authors:** Charlotte Lepleux, Aurélie Marie-Brasset, Mihaela Temelie, Marion Boulanger, Émilie Brotin, Mary B. Goldring, Christophe Hirtz, Guillaume Varès, Tetsuo Nakajima, Yannick Saintigny, Diana Savu, François Chevalier

**Affiliations:** 1 LARIA, iRCM, François Jacob Institute of biology, DRF-CEA, Caen, France; 2 UMR6252 CIMAP, CEA - CNRS - ENSICAEN - Université de Caen Normandie, Caen, France; 3 Department of Life and Environmental Physics, Horia Hulubei National Institute of Physics and Nuclear Engineering, Reactorului 30, P.O. Box MG-6, 077125 Magurele, Romania; 4 ImpedanCELL Platform, Federative Structure 4206 ICORE, Normandie Univ, UNICAEN, Inserm U1086 ANTICIPE « Interdisciplinary Research Unit for Cancer Prevention and Treatment », Biology and Innovative Therapeutics for Ovarian Cancers group (BioTICLA), Comprehensive Cancer Center F. Baclesse, 14000 Caen, France; 5 Research Division, Hospital for Special Surgery and Weill Cornell Medical College, New York, NY USA; 6 University of Montpellier, LBPC/PPC, IRMB, CHU de Montpellier, 34000 Montpellier, France; 7 Cell Signal Unit, OIST, Onna-son, Okinawa, Japan; 8 Dept. of Radiation Effects Research, NIRS, QST, Chiba-shi, Japan

**Keywords:** Chondrosarcoma, Radiation-induced bystander effect, Chondrocyte, High-LET, Low-dose irradiation

## Abstract

While the dose-response relationship of radiation-induced bystander effect (RIBE) is controversial at low and high linear energy transfer (LET), mechanisms and effectors of cell-to-cell communication stay unclear and highly dependent of cell type. In the present study, we investigated the capacity of chondrocytes in responding to bystander factors released by chondrosarcoma cells irradiated at different doses (0.05 to 8 Gy) with X-rays and C-ions. Following a medium transfer protocol, cell survival, proliferation and DNA damages were quantified in bystander chondrocytes. The bystander factors secreted by chondrosarcoma cells were characterized. A significant and major RIBE response was observed in chondrocyte cells (T/C-28a2) receiving conditioned medium from chondrosarcoma cells (SW1353) irradiated with 0.1 Gy of X-rays and 0.05 Gy of C-ions, resulting in cell survivals of 36% and 62%, respectively. Micronuclei induction in bystander cells was observed from the same low doses. The cell survival results obtained by clonogenic assays were confirmed using impedancemetry. The bystander activity was vanished after a heat treatment or a dilution of the conditioned media. The cytokines which are well known as bystander factors, TNF-α and IL-6, were increased as a function of doses and LET according to an ELISA multiplex analysis. Together, the results demonstrate that irradiated chondrosarcoma cells can communicate stress factors to non-irradiated chondrocytes, inducing a wide and specific bystander response related to both doses and LET.

**Electronic supplementary material:**

The online version of this article (10.1007/s12079-019-00515-9) contains supplementary material, which is available to authorized users.

## Introduction

Chondrosarcoma is the third most common primary malignancy of bone after myeloma and osteosarcoma. It corresponds to a bone tumor with a cartilaginous differentiation, and a characteristic extracellular matrix (Evans et al. 1977). This kind of tumor responds poorly to chemotherapy and conventional radio-therapy, *ie* X-rays / other photons irradiations (Moussavi-Harami et al. 2006). In a majority of cases, surgical ablation is the most effective treatment, however, radiation-therapy or chemotherapy are necessary in inoperable or incompletely resected tumors (Dai et al. 2011; Mery et al. 2018). The resistance to radiation therapy is generally believed to be due to the poor vascularity, a low oxygen tension and an abundant extracellular matrix, but the mechanisms underlying that resistance are still unclear (Moussavi-Harami et al. 2006). Chondrosarcoma is now defined as one of the tumor in the first line to be treated by light ions hadrontherapy when this technology is available (Hug et al. 1999; Schulz-Ertner et al. 2007; Uhl et al. 2014; Feuvret et al. 2016; De Amorim Bernstein and DeLaney 2016). Indeed, hadrontherapy with carbon ions (C-ions) presents three majors advantages (Suzuki et al. 2000; Jiang 2012; Walenta and Mueller-Klieser 2016; Durante and Debus 2018) when compared with conventional radio-therapy (X-rays). First, the physics of accelerated particles allows a main dose deposition at the end of the beam track i.e. Bragg peak, reducing the dose in healthy tissues before the tumor, increasing the dose within the tumor and preventing tissues exposition after the tumor. The second advantage of C-ions irradiation is related to the relative biological effect (RBE) of such particle, which allow for the same dose deposit within the tumor to an increased biological effect. For the same physical dose, C-ions are described to induce at least 2.5 to 3 times more cell death, compared to X-rays (Suzuki et al. 2000). The third advantage of C-ions corresponds to the physical accuracy of accelerated particles, allowing a higher irradiation precision of the tumor volume. Even with last generation irradiation machines (pencil beam scanning, or cyber-knife), X-rays presents a penumbra around the irradiation beam, reducing the exactness of the irradiation plan. According to these three advantages, C-ions should be used more often in the treatment of cancer, especially against cancer resistant to X-rays. But this kind of treatment platform is not yet fully developed, especially in Europe, and a lot of studies in radiobiology are still needed to allow such treatment (Walenta and Mueller-Klieser 2016). Over the past two decades, considerable evidence has accumulated showing that irradiations can induce a biological response in non-irradiated cells that are in proximity to irradiated cells (Marín et al. 2015). This biological effect, named “bystander effect”, is mainly dependant of the cell type, and treatment (irradiation quality, dose, time of contact …). This bystander effect is defined *(i)* to occur in close proximity to irradiated cells, *(ii)* to induce a biological response in non-irradiated cells, and *(iii)* this effect induces a cellular response typically associated with direct radiation exposure. While hadrontherapy allows a better precision of the radiation towards the tumor, intercellular communication triggered by the irradiated damaged cells could occur, counter-balancing such physical accuracy of accelerated ions by a biological imprecision which may represent an important cause for radiation side-effects. Despite numerous studies on bystander effects, the mechanisms underlying this cellular response and their physiological role are not well understood and more studies are required to elucidate the real consequences of a bystander effect within and outside the irradiated area (Chevalier et al. 2014).

Here, we aimed to analyse the targeted and non-targeted effects of accelerated ions/X-rays in a context of chondrosarcoma radiotherapy. We decided to use the chondrosarcoma cell line SW1353, which previously showed its capacity in emitting bystander factors (Wakatsuki et al. 2012), and the chondrocyte cell line the T/C28a2, which presents characteristics of authentic human chondrocytes, with a production of several cartilage-specific extracellular matrix proteins (Kokenyesi et al. 2000; Nieminen et al. 2005; Lago et al. 2008; Wang et al. 2011). Some of these specific markers are relevant for radio-biological studies, such as the modulation of MAPK, Erk1/2, p38, and JNK signalling in response to IL-1β (Nieminen et al. 2005) and the expression of the cartilage-specific transcription factor SOX-9 in the transcription regulation of cartilage-specific genes, including COL2A1 and AGRN (Finger et al. 2003).

The main objectives of this study were the characterization of direct effects of C-ions and X-rays irradiation on chondrocytes and compare this effect with a potential bystander effect, observed by transferring the conditioned medium from irradiated chondrosarcoma cells to non-irradiated chondrocytes. Several end-points were analysed (clonogenic survival, proliferation, micro-nuclei formation) and allowed to characterize the irradiation and bystander signatures of chondrocytes. The bystander factors were analysed and some candidates, potentially responsible for these stresses, were proposed.

## Materials and methods

### Cell culture

The chondrosarcoma cell line SW1353, (CLS Cell Lines Service GmbH, Eppelheim, Germany) was initiated from a primary grade II chondrosarcoma of the right humerus from a 72 years old female Caucasian. The immortalized human juvenile chondrocyte cell line, T/C28a2 was obtained from the laboratory of Professor Mary B. Goldring, Hospital for Special Surgery, Weill Medical College of Cornell University (New York, New York.). Briefly, T/C-28a2 cells (Finger et al. 2003; Otero et al. 2012), were initiated by transfecting primary cultures of costal cartilage from a 15-year-old female with a retroviral vector expressing simian virus SV40 large T antigen. The immortalization was subsequently obtained by switching genes involved in cell cycle control, such as P53 and RB (Benoit et al. 1995; Finger et al. 2003). The two cell lines were progressively adapted and cultured in the same culture medium, Minimum Essential Medium Eagle (MEM, M5650, Sigma-Aldrich), supplemented with 5% fetal calf serum, 2 mM L-glutamine and 1% antibiotics (Penicillin-Streptomycin solution, Sigma-Aldrich). All experiments were performed in humidified atmosphere with 5% CO2 and physioxia conditions with 2% O_2_ at 37 °C, in a Heracell™ 150i Tri-Gas incubator.

### Irradiation

For X-rays irradiations a tube tension of 225 kV was used on the Pxi XradSmart 225cX irradiator. At the medium position of the sample holder, in case of low doses irradiations (0,05 to 0,2 Gy), an intensity of 1 mA corresponding to a dose rate of 0,2 Gy/min was selected, and in case of doses between 0,5 and 8 Gy an intensity of 10 mA corresponding to a dose rate of 2 Gy/min was selected.

In case of light ions irradiation and X-rays, all doses for targeted effects are expressed as physical doses in Gy (no RBE included) as proposed by (Kamada et al. 2015); for non-targeted effect on chondrocyte bystander cells, all doses are expressed as “Gy^BY^”, as related to the dose of initially irradiated chondrosarcoma cells.

For carbon ions exposure, two irradiation facilities were used (GANIL and HIMAC) and for each experiment, the origin of the beam will be indicated. More precisely, some irradiations were performed with the GANIL facility (Caen, France), using the IRABAT beam line, according to (Durantel et al. 2016). For these experiments, we selected two LET, in order to mimic the clinical LET during hadrontherapy. The first LET corresponded to the plateau region of healthy tissues before the tumor (LET of irradiation of T/C-28a2 cells = 28 keV/μm), and the second LET corresponded to the SOBP region of the tumor (LET of irradiation of SW1353 cells = 73 keV/μm). The two LET were obtained with a native ^12^C C-ion beam of 95 MeV/A with or without a PMMA device inserted between the exit of the beam and the sample holder: without PMMA (native beam), the LET was 28 keV/μm (2 Gy = 4.46 × 10^7^ particles/cm^2^); and using a 16.9 mm thickness PMMA (degraded beam), the LET was 73 keV/μm (2 Gy = 1.71 × 10^7^ particles/cm^2^). Some repetitions of experiments were performed, when possible, using a C-ions beam at the Heavy Ion Medical Accelerator in Chiba (HIMAC) of the National Institute of Radiological Sciences (NIRS, Chiba, Japan), at room temperature. It concerned the clonogenic assays and micronuclei analysis. C-ions were accelerated with an initial energy of 290 MeV/A and cells were irradiated at the center of a 6 cm spread out Bragg peak (SOBP) region, with an average LET of 50 keV/μm.

### Clonogenic assays of irradiated T/C-28a2 cells

This method was used to screen the sensitivity of cells to different radiation qualities. For this approach, cells at confluency were irradiated in T25 cm^2^ flasks. A sham irradiated control was performed to evaluate the plating efficiency, it represents the 0 Gy sham control. After irradiation, the medium was changed immediately, and then the flasks were placed back in the incubator for at least 24 h. Cells are then harvested and re-plated with appropriate dilutions in the multi-wells plates. A set of 6-wells plates were used with two plating densities, in order to reach about 100 and 1000 clones per well in control samples. After an incubation period of at least 10 days, colonies were stained with a crystal violet solution (0.3% *w*/*v* crystal violet in 20% *v*/v ethanol). Colonies composed of at least 50 cells were counted visually with a stereomicroscope. The results are expressed as a percentage of control un-irradiated cells. A linear-quadratic model of cell survival was used to fit the results obtained with X-Rays irradiation (Rutz et al. 1991) according to the equation: SF(Dose) = exp. (Alpha*Dose + Beta*Dose^2). A linear model of cell survival was used to fit the results obtained with C-ions irradiation according to the equation: SF(Dose) = exp. (a*Dose). All curves were fitted with a dedicated tool for Clonogenic Survival Calculation, the CS-cal software (www.oncoexpress.de).

### Clonogenic assay of bystander T/C-28a2 cells

**Fig. 1:**
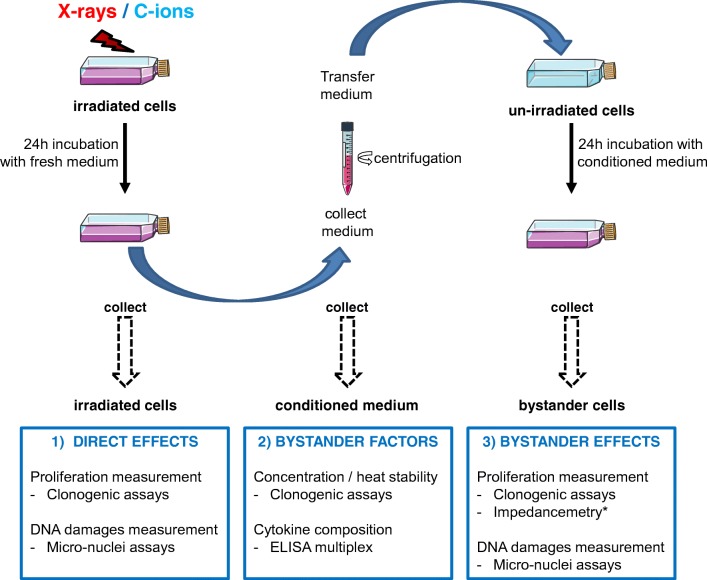
Schematic representation of experiments followed for the medium transfer protocols. Cells were irradiated at confluency in T25 flasks with X-rays or C-ions. Immediately after irradiation, the medium was changed with fresh new medium and these irradiated cells were incubated for 24 h. Then, cells were collected for clonogenic and micro-nuclei assays, and the conditioned medium was centrifuged and collected. This conditioned medium was then characterized for cytokine composition or concentration / heat stability; or transferred to non-irradiated cells for 24 h in T25 flasks with the same cell density. Then, cells were collected for clonogenic and micro-nuclei assays. * in case of impedancemetry analysis, the experiment was not performed on flasks but with specific electrodes plates. Un-irradiated cells were first cultured in these plates, and after 24 h, the medium was changed with the conditioned medium as described before and the cellular index was followed during 4 days

This experiment was used to estimate the bystander effect against cell survival with a “medium transfer” protocol from irradiated cells to non-irradiated cells. For this protocol, irradiated SW1353 cells and T/C-28a2 bystander cells were plated in T25 cm^2^ flasks at confluency. Immediately after irradiation, the medium of irradiated flasks was changed with fresh medium and after 24 h in contact with irradiated SW1353 cells (to allow the bystander factors to be released), this medium was collected (Fig. 1). The condition medium was then centrifuged (2000 g) and transferred in flasks of the same size (T25 cm^2^) containing bystander T/C-28a2 cells at confluency. Bystander cells were kept in contact with the conditioned medium for 24 h and then harvested and re-plated at low density in a set of 6 wells plates, as described in the previous paragraph concerning clonogenic assays for direct effect study. The results are expressed as a percentage of control cells receiving the medium from un-irradiated cells.

### Bystander factors analysis using clonogenic assay

In the case of the dilution study of the bystander medium, the same clonogenic assay protocol (as for Bystander effect study previously described) was used, but the conditioned medium was diluted with fresh medium at 50%, 25% and 10% before transferring to culture of Bystander cells. A control (100%) with non-diluted conditioned medium was performed. In the case of the heat treatment study of the bystander medium, before transferring the conditioned medium to bystander cells and analyze survival rate with the clonogenic assay protocol, the medium was heated at 70 °C and 95 °C. A control with un-heated conditioned medium was performed. Experiments with TNF-α (Sigma-Aldrich) were performed with serial dilution of TNF-α in culture medium.

### Real-time cell analysis (Impedancemetry)

Bystander-mediated cytotoxicity was monitored with the Real-Time Cell Analyzer Multi-Plate (RTCA MP) Instrument, using the xCELLigence System (ACEA, Ozyme, France). This system monitors cellular events in real time by measuring electrical impedance across interdigitated micro-electrodes integrated on the bottom of tissue culture E-plates VIEW. These electrodes measure CI (Cell Index) based on impedance. CI correlates with the area of cells attached to the bottom of the plate. The Cell Index (CI) values are displayed in the plot. Briefly, chondrosarcoma cells (SW1353) were irradiated at confluency in T25 cm^2^ flasks and bystander cells (T/C-28a2) were plated in an E-Plate VIEW 96 (7500 cells per well) and placed onto the RTCA MP located inside a tissue culture incubator. Immediately after irradiation, the medium of irradiated T25 flasks was changed with fresh medium and after 24 h in contact with irradiated cells, this medium was collected, centrifuged (2000 g) and transferred in wells (E-Plate View 96) already containing bystander cells. Cells were left to grow for 6 days with conditioned medium and impedance was continuously measured. Standard deviations of well replicates were analyzed with the RTCA 2.1.0 Software.

### In vitro micronuclei analysis

T/C28a2 cells were cultured on glass coverslips in 24-well plate with 500 μL medium. For the study of directly irradiated cells, cytochalasin B (Cyt B) was added at 3 μg/ml 4 h following irradiation and then incubated during 20 h. Cells were then washed once with PBS and fixed with methanol – acetic acid (9:1) and stored at 4 °C for 2 h. After rinsing with PBS, the Antifade Reagent proLong Gold with DAPI (Molecular Probes), was used and MN were observed by fluorescence microscopy. The results were analyzed by calculating the binucleated micronucleted cells frequency as the number of binucleated cells containing one or more micronuclei per 1000 binucleated cells. Micronuclei were identified according to (Countryman and Heddle 1976): diameter less than 1/3 of the main nucleus, non-refractility, not touching the nucleus, and the same color as the nucleus or lighter. Independent experiments were performed with three wells per each treatment condition. In the case of bystander studies, T/C28a2 cells were first incubated during 24 h with conditioned medium of SW1353 irradiated cells (same protocol of preparation as paragraph “Clonogenic assay for Bystander effect study”) and then, bystander cells were treated with cytochalasin B as described within this paragraph for directly irradiated cells.

### Cytokines study in the bystander supernatant

The V-PLEX Human Pro inflammatory Panel II 4 plex (ref K15053D-1) was used and allowed the quantification of 4 cytokines (IL-1β, IL-6, IL-8 and TNF-α) by ELISA multiplex with MSD kit. SW1353 cells at culture confluency were irradiated in T 12.5 cm^2^ flasks, the medium was changed immediately after irradiation and replaced with fresh medium (without serum); 24 h after irradiation the supernatants were collected, centrifuged 10 min at 3000 rpm supplemented with anti-proteases and anti-phosphatases. The samples were stored at −80 °C until analysis according to MSD instructions.

### Statistical analysis

The statistical analysis was performed using the statistical module of the Origin software (V 6.0), by a t-test (two populations) with an independent type and a 0.05 significant level. Data set were considered as significantly different when *p* < 0.05 (*) and *p* < 0.01 (**).

## Results

### Cell survival

Cell survival was analysed on directly irradiated and bystander T/C-28a2 cells, receiving conditioned medium from irradiated cells. A clonogenic assay protocol was set up to study and compare both cellular responses, taking into account the time for the secretion of bystander factor by irradiated cells and the time for the reception of these factor by non-irradiated / bystander cells. In both cases, the same incubation time (24 h) was selected and the same clonogenic protocol was done after treatment (Fig. 1). Since this protocol included a medium transfer, a first step of this study consisted in adapting both cell lines to the same medium condition, in order to eliminate the stress of a modification of the medium composition itself.

**Fig. 2:**
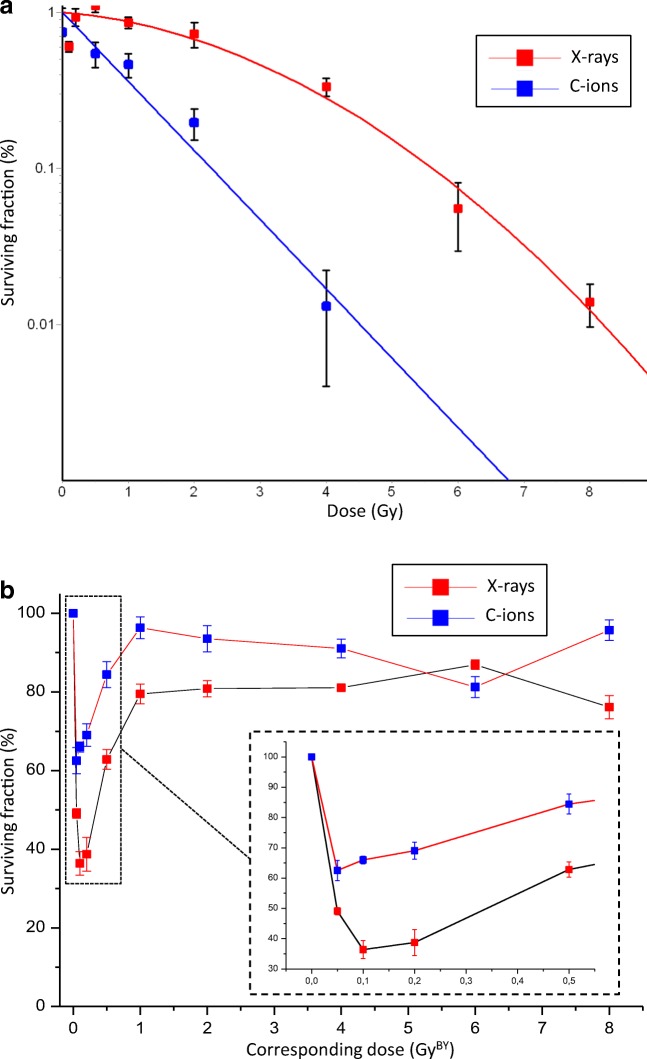
Cell survival of T/C-28a2 chondrocytes. **a** surviving fraction of cells directly exposed to 225 KV X-rays (red) and 28 keV/μM C-ions (blue); all values were normalized (%) against a sham-irradiated control sample. **b** surviving fraction of cells exposed to conditioned medium from SW1353 cells irradiated with 225 KV X-rays (red) and 73 keV/μM C-ions (blue); the corresponding dose (GY^BY^) matched to the irradiation doses of SW1353 cells; all values were normalized (%) against a control sample receiving a conditioned medium from sham-irradiated SW1353 cells. Values are means ± SEM for *n* = 3 from at least 2 independent experiments

Cell survival was first analysed on directly irradiated cells (Fig. 2a). The chondrocyte cell line was irradiated with X-rays (red line) and C-ions (blue line) at different doses. As expected, C-ions irradiations decreased the surviving fraction when compared with X-rays. As specified in the Materials and Methods, a specific LET (28 keV/μm) was selected for C-ions irradiation of T/C28-a2 cells, corresponding to the LET of the plateau (healthy tissue). A relative biological effect (RBE) can be calculated using the cell survival parameters (Table 1). Two RBE of 2.49 and 3.58 were calculated using the D_10_ and the D_37_ values, respectively.

**Table 1:** Cell survival parameters of T/C-28a2 cells (fitted from curves of Fig. 2a)

	D10^a^	D37^b^	SF2^c^	RBE (D10)^d^	RBE (D37)^e^
X-Rays 225 KV	5.60	3.47	0,67	/	/
C-ions 28 keV/μm	2,25	0.97	0,12	2.49	3.58

^a^ The D10 dose gives a surviving fraction of 0.1

^b^ The D37 dose gives a surviving fraction of 0.37

^c^ The SF2 fraction is observed at a 2Gy irradiation

^d^ RBE (D10) values are calculated as (D10 X-Rays) / (D10 C-ions)

^e^ RBE (D37) values are calculated as (D37 X-Rays) / (D37 C-ions)

X-Rays irradiation: Linear-quadratic model

SF(Dose) = exp. (Alpha*Dose + Beta*Dose^2)

SF(0) = −0,1812

Alpha = −0,0831

Beta = −0,0583

Alpha/Beta - ratio = 14,249

C-ions irradiation: Linear model:

SF(Dose) = exp. (a*Dose)

SF(0) = 0,2998

a = −10,212

Knowing that SW1353 cells were able to produce bystander stress factor following irradiation (Wakatsuki et al. 2012), we analysed the capacity of the corresponding healthy cells, *ie* chondrocyte cells (T/C28-a2) to receive and respond to such factors. A medium transfer protocol was used for this analysis and the surviving fraction of T/C28-a2 cells receiving this medium was analysed (Fig. 2b). It is important to notice that no irradiated media were transferred to bystander cells. Indeed, for this experiment, SW1353 cells were irradiated with X-rays and C-ions using a LET of the SOBP region (73 keV/μm), and immediately after irradiation, cell culture media were changed with fresh new media (Fig. 1). SW1353 cells were irradiated with doses ranging from 0.05 to 8 Gy. The highest effect was observed at the lowest doses (inset panel of Fig. 2b). Indeed, when considering X-rays irradiation (in red), the minimum surviving fraction (36%) of T/C28-a2 bystander cells was obtained when the medium of SW1353 cells irradiated at 0.1 Gy was transferred (corresponding dose of 0.1 Gy^BY^). The same phenomenon was observed with C-ions irradiations, at a lower amplitude, in that case, the minimum surviving fraction of T/C28-a2 bystander cells was about 62%, when the medium of SW1353 cells irradiated at 0.05 Gy was transferred. This bystander effect was maximum at low doses and reached a plateau from 1 Gy for both X-rays and C-ions corresponding doses. The tendency of this plateau was about 80% of survival with X-rays and about 90% with C-ions, with some irregularities at higher doses (6 Gy^BY^).

### Cell proliferation

**Fig. 3:**
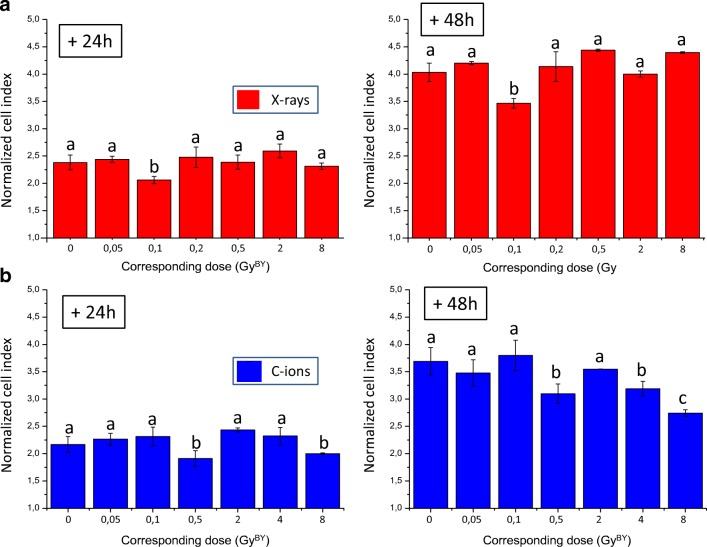
Normalized cell index (impedance-based) of cells exposed to conditioned medium from SW1353 cells irradiated with 225 KV X-rays (A, in red) and 73 keV/μM C-ions (B in blue) during 24 h and 48 h; the corresponding dose (GY^BY^) matched to the irradiation doses of SW1353 cells

To further analyze and characterize this bystander effect, we analyzed the capacity of the conditioned medium from irradiated SW1353 cells to modify bystander cell viability and proliferation. A real-time cell analysis based on impedancemetry (xCELLigence System) of the bystander response of T/C28-a2 cells was performed. A complete picture of the cellular index of T/C28-a2 cells was added as supplementary data. The cells were first seeded in the system microplate, and 24 h later, the conditioned medium from SW1353 irradiated cells was added. This time point was used to normalize all replicates (t0) and in the linear range of the cell growth, at t + 24 h and t + 48 h, the corresponding cell index were reported and compared between the corresponding doses in Gy^BY^ (Fig. 3). When considering X-rays (in red), a significant decrease of the cellular index was observed with 0.1 Gy^BY^ from 24 h to 48 h. All the other doses were not significantly different. In case of C-ions (in green), a significant reduction of the cellular index of T/C28-a2 cells was observed when SW1353 cells were irradiated at 0.5 and 8 Gy from 24 h to 48 h.

### DNA damage

**Fig. 4:**
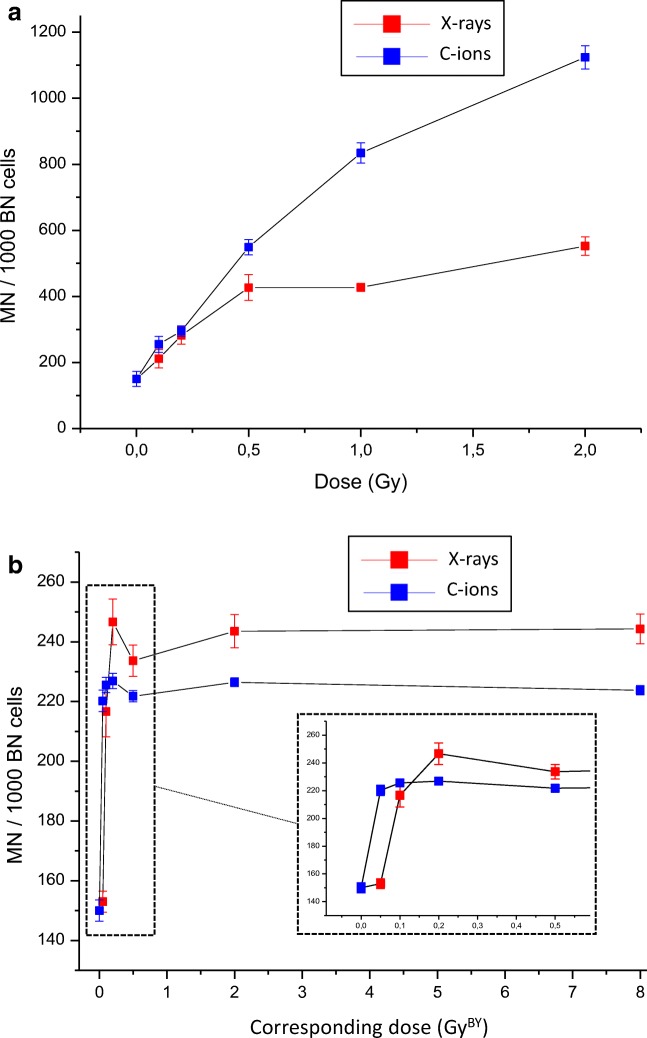
Micronucleus induction in T/C-28a2 chondrocytes. **a** number of micronuclei per 1000 binucleate cells directly exposed to 225 KV X-rays (red) and 28 keV/μM C-ions (blue). **b** number of micronuclei per 1000 binucleate cells exposed to conditioned medium from SW1353 cells irradiated with 225 KV X-rays (red) and 73 keV/μM C-ions (blue); the corresponding dose (GY^BY^) matched to the irradiation doses of SW1353 cells. Values are means ± SEM for n = 3 from at least 2 independent experiments

Micro-nuclei induction was first quantified in directly irradiated T/C28-a2 cells (Fig. 4a). As attempted, a dose – dependent induction of micro-nuclei was observed with X-rays (red) and C-ions (blue), but with a higher induction after C-ions exposure. A generic RBE (MN C-ions / MN X-rays) was estimated and corresponded to 1.95 (at 1 Gy) and 2.03 (at 2 Gy). Micro-nuclei induction was then analyzed in bystander cells (T/C28-a2) receiving the medium of irradiated cells (SW1353) and compared between X-rays and C-ions (Fig. 4b). In case of X-rays, the number of MN increased in bystander T/C28-a2 cells from 0.1 Gy^BY^ and reached a plateau at 0.2 Gy^BY^ until 8 Gy^BY^ with about 240 MN / BN cells (inset panel of Fig. 4b). In case of C-ions, the number of MN increased in bystander T/C28-a2 cells from 0.05 Gy^BY^ and reached a plateau at 0.1 Gy^BY^ until 8 Gy^BY^ with about 225 MN / BN cells.

### Stability and composition of bystander medium

**Fig. 5:**
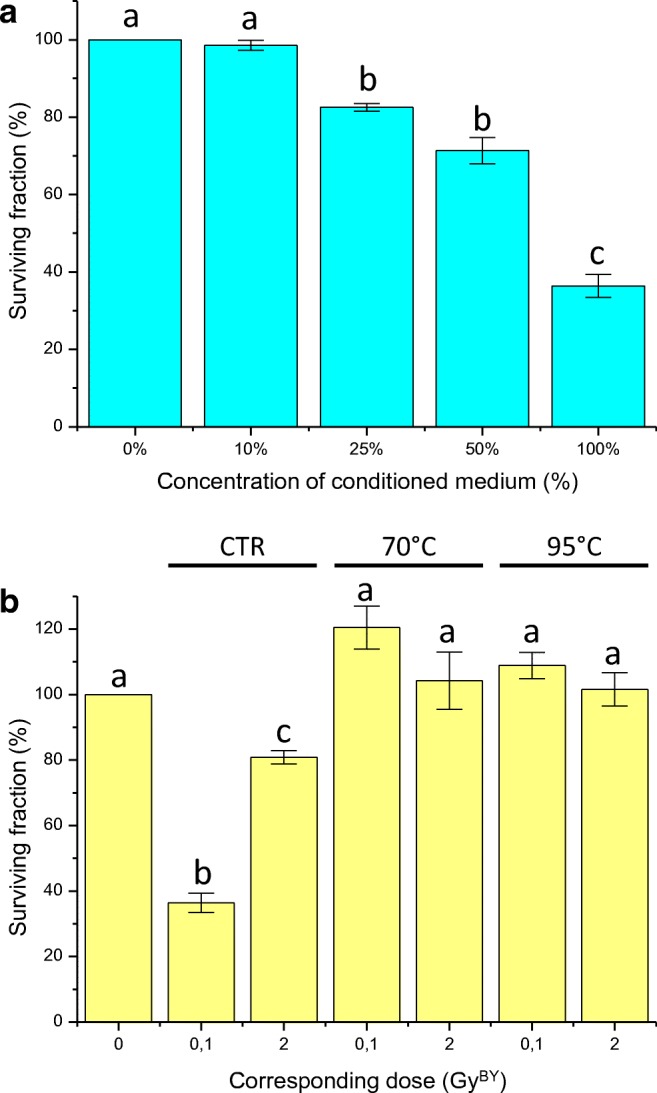
**a** Cell survival of T/C-28a2 chondrocytes exposed to different dilutions (%) of conditioned medium from SW1353 cells irradiated with 0.1 Gy of 225 KV X-rays. **b** cell survival of T/C-28a2 chondrocytes exposed to conditioned medium from SW1353 cells irradiated with 0.1 and 2 Gy of 225 KV X-rays, and pre-heated at 25 °C (control), 70 °C and 95 °C. The corresponding dose (GY^BY^) matched to the irradiation doses of SW1353 cells. Values are means ± SEM for n = 3 from at least 2 independent experiments

A cell survival assay was performed with T/C28-a2 bystander cells using a diluted conditioned medium from irradiated SW1353 cells (Fig. 5a). When 10% of conditioned medium was used (diluted with 90% of fresh medium), no effect was observed. When 25% of conditioned medium was used (with 75% of fresh medium), a reduction of the survival rate of bystander cells was observed with 82% of surviving fraction. And when half of the conditioned medium was used, almost half of the effect (72%) was observed when compared with the un-diluted conditioned medium (36%). In addition, heating experiments were performed with the same strategy, and we observed a heat sensitivity of the conditioned medium from 70 °C. Indeed, when the medium was heated at 70 °C and at 95 °C, no bystander effect was observed on T/C28-a2 cells (Fig. 5b).

**Fig. 6:**
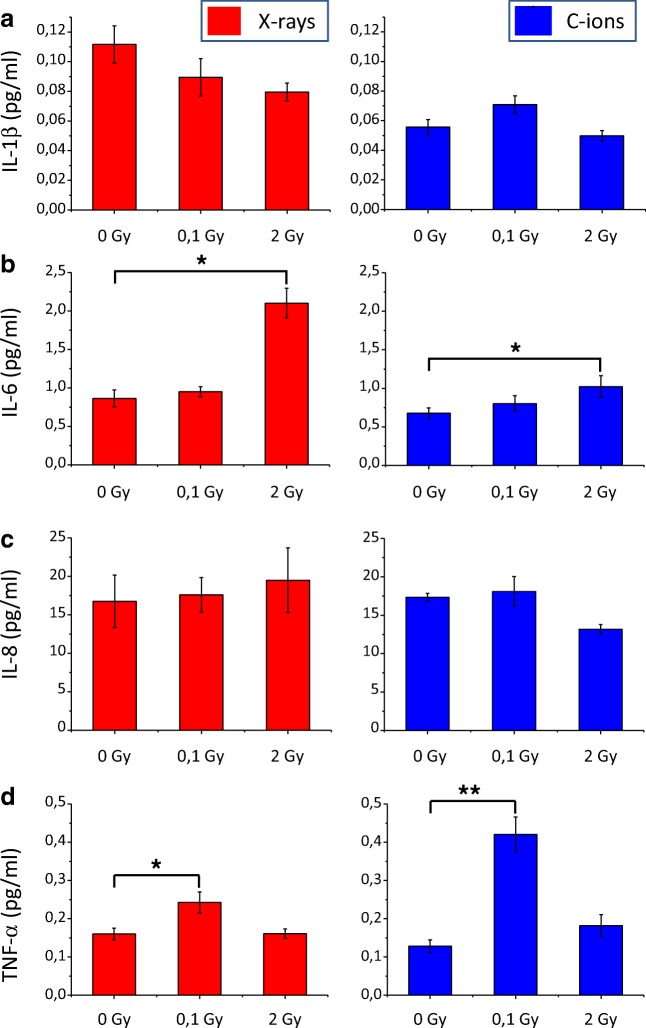
Expression (reported to sham-irradiated control samples) of factors secreted in conditioned medium from SW1353 cells irradiated with 225 kV X-rays (red) and 73 keV/μm C-ions (blue) by ELISA multiplex quantification (pg/ml) of IL-1β (**a**), IL-6 (**b**), IL-8 (**c**) and TNF-α (D) factors. Values are means from 3 independent experiments. Data were represented as the mean of three independent replicates and the variation was evaluated with the standard deviation using a 95% confidence interval. Statistics were performed using a t-test (two independent population) and data were considered as significantly different when *p* < 0.05 (*) and *p* < 0.01 (**)

Some of the factors susceptible to be secreted by irradiated cells were analyzed by ELISA multiplex (Fig. 6). Using the ELISA MSD technology, we analyzed 4 cytokines, and only IL-6 and TNF-α were significantly increased when compared with un-irradiated samples. TNF-α concentration was increased 3.2 and 1.5 times in conditioned medium of SW1353 cells irradiated with 0.1 Gy of C-ions and X-rays, respectively. When added to fresh medium in the same experimental conditions, TNF-α was able to reduce significantly the surviving fraction of T/C28-a2 cells (Sup Fig. 1). IL-6 concentration was increased 2.4 and 1.5 times in conditioned medium of SW1353 cells irradiated with 2 Gy of X-rays and C-ions, respectively. No modulation of IL-1β and IL-8 were observed with these tests in our conditions.

## Discussion

One of the major issues of cancer treatment is the balance between enhancing the impact on the tumor tissue and a preservation of healthy tissues. This is a reason for the development of new machines and technologies, including pencil beam scanning, intensity modulation and hadron-therapy. Unfortunately, most of these progresses were based on physical characteristics of irradiation beams, with only weak estimations of radiobiological response of cells involved. In the past 25 years, multiple lines of evidence emerged in demonstrating the presence of biological responses in non-irradiated cells located close to the irradiated volume. Following the first study in 2012 (Wakatsuki et al. 2012), we analyzed here the bystander effect of chondrosarcoma cells irradiated with X-rays and C-ions, using different approaches since this effect is highly dependent on the end-points studied (Chevalier et al. 2014).

Knowing that the bystander effect is described to produce biological effects which were typically observed within the irradiated volume, we first analyzed the effect of directly irradiated (targeted) cells. T/C-28a2 cells were irradiated with X-rays and C-ions at different doses, and clonogenic assays were performed to analyze the survival rate of these cells. To our knowledge, this immortalized chondrocyte cell line was irradiated for the first time and according to the survival fitted curves (Fig. 1a) and micronuclei assays (Fig. 4a), these cells displayed a characteristic response to X-rays and C-ions irradiation. A RBE of 2.49 (Table 1) in the range of previous studies (Suzuki et al. 2000) was obtained from D10 values and a RBE of 1.95 was obtained from MN at 1Gy.

Following the evaluation of these basic radio-biological values, we then analyzed the bystander response of T/C-28a2 cells, using a medium transfer protocol. The chondrosarcoma cell line SW1353 was irradiated with X-rays and C-ions, and the conditioned medium from these cells was then transferred to un-irradiated T/C-28a2 cells (Fig. 1). For these bystander experiments, SW1353 cells were irradiated with doses ranging from low doses (0.05 Gy) until high dose (8 Gy) with both irradiation qualities. However, too low doses of C-ions could generate irradiation homogeneity issues (Durantel et al. 2016). In our conditions of C-ions, the dose of 0.05 Gy corresponded to a ion flux of 2,85 10^4^ particles cm^−2^ s^−1^ and an estimated dose rate of 0.2 Gy min^−1^, in the calibration range of the IRABAT line of GANIL facility (Durantel et al. 2016). With this flux, the dose 0.05 Gy was reached after 15 s irradiation with a fluency of 4.28 10^5^ C-ions cm^−2^ at a LET of 73 keV/μm. Considering the surface of the flasks (25 cm^2^) and the cell number (about 2 10^6^ cells/flasks at confluency), we can estimate that each cell was hit by an average of 5 ions at the dose of 0.05 Gy. Although this fluency is fairly low, it remains particularly accurate and homogeneous in our dosimetry calibration and set-up (Durantel et al. 2016; Boissonnat et al. 2017). As a matter of fact, such dose is certainly the lowest dose applicable for radio-biology experiments in GANIL facility.

T/C-28a2 cells receiving the medium of SW1353 irradiated cells displayed a bystander response depending on the dose, the irradiation quality and the endpoint studied. It is significant to notice that a similar bystander response was observed when using clonogenic assays or MN assays. Using clonogenic assays, the lowest survival fraction was measured at 0.1 Gy^BY^ with X-rays and 0.05 Gy^BY^ with C-ions (Fig. 2b). Using MN assays, the significative induction of MN started from 0.05 Gy^BY^ with C-ions and from 0.1 Gy^BY^ with X-rays and for doses higher than 0.2 Gy^BY^, X-rays induced more MN than C-ions (Fig. 4b). In the case of MN assays, our results are very similar to the results from (Wakatsuki et al. 2012) using fibroblasts as bystander cells and SW1353 cells as irradiated cells with an induction of MN in bystander cells at low dose, and a plateau for higher doses. But the major difference between clonogenic assays or MN assays was observed at doses up to 1 Gy^BY^. Indeed, a saturation (plateau) of the bystander effect was noted with MN assays from 0.2 Gy^BY^ on contrary to clonogenic assays where no saturation was observed. At the opposite, a reduction of the bystander effect was then measured from 0.2 to 1 Gy^BY^ with an increase of cell survival until a new saturation phase from 1 Gy^BY^ to 8 Gy^BY^. When investigating the bystander effect by impedancemetry, again, differences were highlighted even if some similarities were scored; the lowest cell index was observed at 0.1 Gy^BY^ with X-rays and at 0.5, 4 and 8 Gy^BY^ with C-ions (Fig. 3). With this last approach, the bystander effect was specifically observed at a single dose of 0.1 Gy^BY^ with X-rays, but with C-ions, several doses can induce a proliferation delay. Considering all these results, we analyzed that clonogenic assays with a medium transfer protocol gave the largest differences between controls and bystander treated cells. In our hands, this protocol was the most sensitive with only 36% of cell survival at a dose of 0.1 Gy^BY^, with X-rays and 62% of cell survival at a dose of 0.05 Gy^BY^, with C-ions. According to these results, to reach the same clonogenic survival with direct effect, cells need to be irradiated with about 3.5 Gy with X-rays and 0.5 Gy with C-ions. Such high effect can be related to the good sensitivity of the clonogenic assays, but it can also be related to the genetic characteristics of the recipient cells. Indeed, T/C-28a2 cells are well documented as a good cellular model for cartilage studies in vitro (Kokenyesi et al. 2000; Finger et al. 2003; Otero et al. 2012). But these cells were modified to allow immortalization, and this could have an impact on the biological response to stress factors. As previously described, the process of immortalization conducts to the loss of several cell cycle controls, including p53 and RB (Benoit et al. 1995). According to several studies, p53 was described to play a major function in the bystander effect in both irradiated (He et al. 2014) and recipient cells (Tomita et al. 2013). In human non-small cell lung cancer H1299 cells expressing wild-type p53 or mutation in the p53 gene, a reduction of the survival fraction was observed as higher (10%) in mutated cells, using co-cultures and X-rays micro-beams (Tomita et al. 2013). Using a cell co-culture system containing normal human hepatocytes and irradiated human lymphocytes bearing wild type p53 and mutant p53, bystander effect was p53-dependent for low LET irradiation, but p53-independent for high LET irradiation (He et al. 2014). The responses to radiation exposure and bystander effect of HCT116 colon carcinoma cells with wild-type and knockout p53 gene were compared in directly exposed and in bystander cells (Widel et al. 2015), p53 was engaged in senescence induction, whereas cells deprived of both alleles of TP53 died predominantly through apoptosis. In mice, the bone marrow of irradiated p53 wild type, but not p53 mutated, produced the inflammatory pro-apoptotic cytokines FasL and TNF-α able to induce p53-independent apoptosis in vitro in non-irradiated p53 mutant bone marrow cells (Lorimore et al. 2013).

Finally, it appears as very difficult to compare our results with previously published studies since the bystander effect is highly dependent on the cell types and mutation status (irradiated and bystander), irradiation doses (low vs high doses), irradiations quality (low vs high LET), bystander protocol (medium transfer, co-culture, shield, micro-beam…) and finally the studied end-points (survival, MN, exosomes, proliferation…) (Azzam et al. 2004; Wideł et al. 2009; Rzeszowska-Wolny et al. 2009; Blyth and Sykes 2011; Marín et al. 2015; Klammer et al. 2015). As an example, compared with low LET, high LET particles were described to reduce plating efficiency, to increase chromosomal damages and oxidation of proteins and lipids in the progeny of co-cultured bystander cells (Buonanno et al. 2011), which is in agreement with our MN assays, but differing with our clonogenic assays.

A way to link some of these studies could be the analysis of bystander effector. The stress factors secreted or transmitted from irradiated cells to non-irradiated – bystander cells are certainly dependent of the cell type, but their characterization can give general information on the cellular pathway impacted, independently to the bystander protocol and end-point studied. In our case, a dilution of the conditioned medium from irradiated chondrosarcoma cells still induced a bystander effect, but with a lower extent when used at 50% and 25%, and the bystander effect was lost when only 10% of the conditioned medium was used (Fig. 5a). Using the same dilution protocol, the bystander effect against HPV-G cells was lost when using 50% of conditioned medium from irradiated HPV-G, HaCAT and SW48 cells (Ryan et al. 2008). Moreover, the conditioned medium from irradiated chondrosarcoma cells appeared as thermo-sensitive, the bystander effect was lost when the medium was heated at 70 °C (Fig. 5b). In case of bystander effectors, using an ELISA multiplex, TNFα was observed as increased significantly at 0.1 Gy with both X-rays and C-ions irradiations (Fig. 6). IL-6 was also observed increased by X-rays in the conditioned medium of irradiated SW1353 cells, but at the dose of 0.1 Gy. The secretion of pro-inflammatory cytokines, such as IL-6, was described to be linked with the primary ATM-NF-κB signaling pathway, as a characteristic aspect of persistent DNA damage signaling (Rodier et al. 2009; Ivanov et al. 2010; Hei et al. 2011). Since a dilution of the conditioned medium decreased the bystander effect, only a secretion of a factor (increased in concentration) in response to irradiation can explain such effect. We could then hypothesize that TNFα may responsible (at least partially) of the bystander effect observed at low dose (0.1 Gy) and IL-6 at higher doses (2 to 8 Gy). Indeed, TNFα alone, diluted in cell culture medium induced a biological effect on chondrocytes (Sup Fig. 1), showing at least a potential and partial role in mediating the bystander effect from irradiated chondrosarcoma cells to recipient chondrocytes. TNF-α was previously described as a central effector of the bystander cellular response, activating MAPK pathways, COX-2 and iNOS expression (Zhou et al. 2005; Hei et al. 2008). In our study, according to the treatment of conditioned medium, the bystander factor appeared as thermo-sensible, which implied certainly a functional 3-dimensional organization / well-structured factor. This is compatible with cytokine effectors, TNF-α and IL-6 present well-defined 3D-structures, allowing the proteins to recognize specifically receptors (Somers et al. 1997; Horiuchi et al. 2010), and this factors is not resistant to high temperature (Kenis et al. 2002). But our results were compatibles with a lot of other cytokines and to the presence of exosomes too. Indeed, micro-vesicles are of growing interest in the transmission of the radiation-induced bystander effect (Jelonek et al. 2016), and these structure can be thermo-sensible (Nguyen et al. 2017) and concentration correlated. These results are promising, but such characterization of the bystander effectors still need further functional experiments on the already identified factors, and as well, other experiments with a larger panel of potentially involved cytokines.

## Conclusion

Collectively, this work brings new data on the prevalence of the bystander effect, especially at low doses. Even using high LET particle, it is technically possible to study the bystander response at doses as low as 0.05 Gy. Chondrosarcoma, which is a cancer type likely to be treatable by hadrontherapy due to an established radio-resistance to X-rays, presents a capacity to produce bystander factors. Our experiments clearly showed that a radio-induced bystander response can be transmitted from irradiated chondrosarcoma cells, to non-irradiated chondrocyte bystander cells. A major impact on chondrocyte survival was observed at low doses, with a higher effect after low LET (X-rays) as compared with high LET irradiation (C-ions). Bystander factors secreted in the conditioned medium were able to reduce proliferation and increase DNA damages at low doses (X-rays and C-ions). Bystander biological activity was missed after dilution and heat treatment of the conditioned medium, and factors such as TNF-α and IL-6 were proposed to contribute to this effect. Additional investigations are still needed to understand the bystander response analyzed in this study, including the potential role of exosomes, p53 status and oxidative stress in the propagation of the effect after irradiation of chondrosarcoma cells. Nevertheless, with this study, we showed for the first time that chondrosarcoma cells and chondrocytes can communicate by radiation-induced mechanisms. These results highlight the significance of taking into account this biological effect in order to preserve the normal and radiosensitive tissues in the close vicinity of the irradiated volume, during radio- and hadron- therapy of chondrosarcoma, even with highly accurate machines.

## Electronic supplementary material

Supplementary figure 1 Cell survival of T/C-28a2 chondrocytes exposed to different concentration of TNF-α in fresh medium. Values are means ± SEM for *n* = 3 from at least 2 independent experiments. (PDF 87 kb)
