# Supplementary figures and images for: Bystander effectors of chondrosarcoma cells irradiated at different LET impair proliferation of chondrocytes
Source: J Cell Commun Signal. 2019 Mar 22;13(3):343–56. doi: 10.1007/s12079-019-00515-9 (PMC6732157; doi:10.1007/s12079-019-00515-9)

Supplementary figure 1

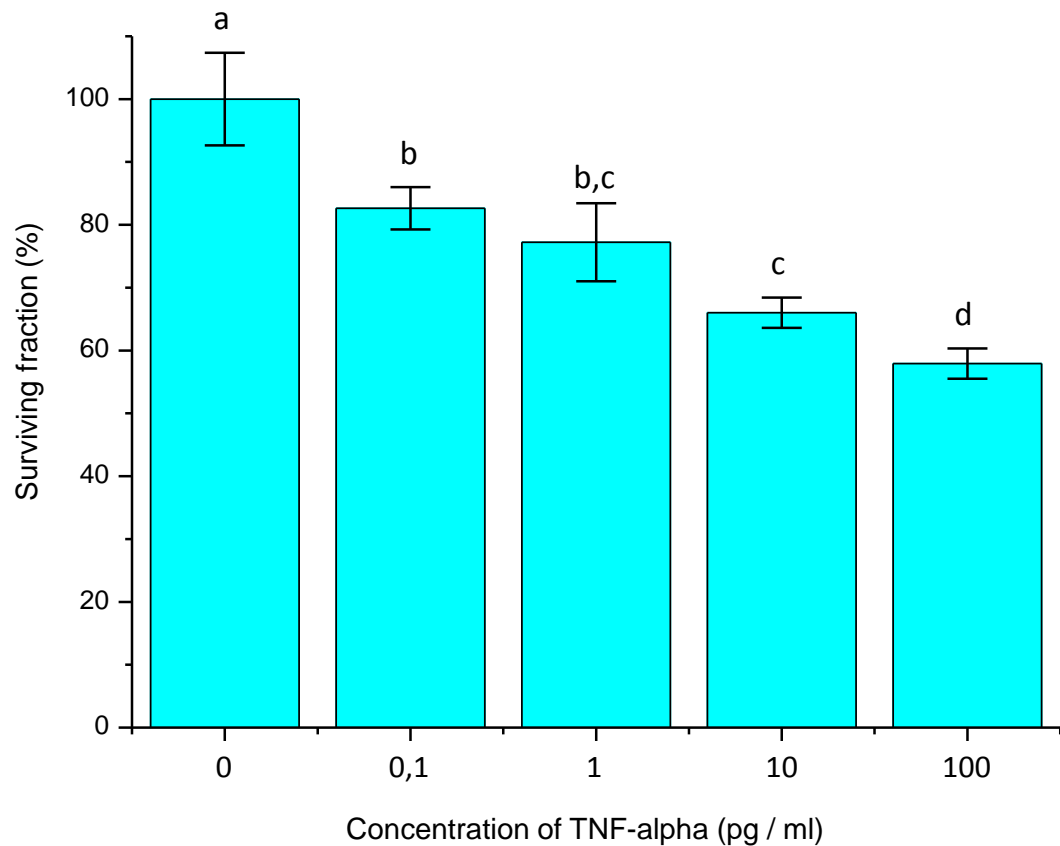

Supplement: Supplementary file 1 — Cell survival of T/C-28a2 chondrocytes exposed to different concentration of TNF-α in fresh medium. Values are means ± SEM for n = 3 from at least 2 independent experiments. (PDF 87 kb) [file 12079_2019_515_MOESM1_ESM.pdf]
